# Von Hippel-Lindau (VHL) Protein Antagonist VH298 Improves Wound Healing in Streptozotocin-Induced Hyperglycaemic Rats by Activating Hypoxia-Inducible Factor- (HIF-) 1 Signalling

**DOI:** 10.1155/2019/1897174

**Published:** 2019-02-17

**Authors:** Shuo Qiu, Yachao Jia, Yunchu Sun, Pei Han, Jia Xu, Gen Wen, Yimin Chai

**Affiliations:** Department of Orthopaedic Surgery, Shanghai Jiao Tong University Affiliated Sixth People's Hospital, Shanghai, China

## Abstract

**Aims:**

The purpose of the present research is to investigate the effects of the VHL protein antagonist, VH298, on functional activities of fibroblasts and vascular endothelial cells and the effects on the wound healing process in a streptozotocin-induced hyperglycaemic rat model.

**Methods:**

HIF-1*α* and hydroxy-HIF-1*α* protein levels in VH298-treated rat fibroblasts (rFb) were measured by immunoblotting, rFb proliferation was detected by the CCK-8 assay, and mRNA levels of related genes were measured by quantitative RT-PCR. In vitro wound healing was simulated by the scratch test; angiogenesis was measured by the human umbilical vein endothelial cell (hUVEC) tube formation assay. VH298 or PBS was locally injected into wounds in rat models with streptozotocin- (STZ-) induced hyperglycaemia, the wound tissues were harvested, and haematoxylin-eosin (HE) and Masson trichrome staining and immunohistochemical processes were conducted.

**Results:**

HIF-1*α* and hydroxy-HIF-1*α* levels increased in VH298-treated rFb, in a time- and dose-dependent manner. Thirty micromolar VH298 could significantly increase cell proliferation, angiogenesis, and gene expression of type I collagen-*α*1 (Col1-*α*1), vascular endothelial growth factor A (VEGF-A), and insulin-like growth factor 1 (IGF-1). The VH298-treated wound had a better healing pattern, activation of HIF-1 signalling, and vascularization.

**Conclusions:**

Taken together, VH298 activated the HIF-1 signalling pathway by stabilizing both HIF-1*α* and hydroxy-HIF-1*α*. VH298 enhanced rFb functions, promoted hUVEC angiogenesis, and accelerated wound healing in the rat model mimicking diabetes mellitus.

## 1. Introduction

Wound healing is much slower in patients with diabetes than in others, the former being more likely to develop postsurgical wound complications even if diabetes is under control [1]. Delayed wound healing in patients with diabetes mellitus (DM) often leads to infection and chronic ulceration and may even result in gangrene and amputation of extremities [2]. Therefore, impaired wound healing in patients with DM is a significant issue, as a heavy burden not only on the patients but also on the health care system. Catrina et al. first noticed that hyperglycaemia impaired HIF-1*α* protection under hypoxia in human diabetic ulcers and pointed out the molecular mechanism connecting hyperglycaemia and hypoxia sensitivity [3], and Mace et al. disclosed that compared to nondiabetic, hypoxia-inducible factor- (HIF-) 1*α* expression was markedly decreased in skin wounds of diabetic mice [4].

HIF-1, a transcriptional regulatory factor, consists of HIF-1*α* and HIF-1*β* subunits. Since HIF-1*β* heterodimerises with other proteins and occurs abundantly, HIF-1*α* protein levels determine HIF-1 transcriptional activity [5]. However, HIF-1*α* is present in very low levels under well-oxygenated conditions; HIF-1*α* is hydroxylated by prolyl hydroxylases (PHD), in which the cosubstrate, *α*-ketoglutarate, is also oxidized and split into CO_2_ and succinate [6]. Hydroxylation of HIF-1*α* is essential for binding to Von Hippel-Lindau (VHL) protein, which recruits an E3 ubiquitin ligase, thereby leading HIF-1*α* into proteasomal degradation [6].

HIF-1 activators have been widely analysed, but almost all have targeted the hydroxylation process; typically, dimethyloxalylglycine (DMOG), a competitive antagonist of *α*-ketoglutarate, and deferoxamine (DFO) inhibit hydroxylases by displacing Fe(II) from their catalytic centre. But the effect of the VHL inhibitor is barely known. Frost et al. [7] were the first to reveal a potent and selective chemical compound, a VHL inhibitor named VH298, which added a new dimension in HIF-targeting therapeutics.

HIF-1 has been shown to upregulate angiogenic and multiple factors and promote wound healing [8, 9]. The wound area in patients with DM is hypoxic, where the HIF-1*α* level is abnormally reduced [10]. Therefore, we hypothesised that increasing the HIF-1*α* level using VH298 could improve wound healing in patients with DM.

## 2. Materials and Methods

### 2.1. Cell Culture

The rFb and hUVEC were purchased from ScienCell (Carlsbad, CA, USA). Briefly, rFb were cultured in fibroblast medium (FM; ScienCell), and hUVEC in endothelial cell medium (ECM; ScienCell), at 37°C with 5% CO_2_ and 95% humidity. Cells from passages 6–8 were used in the experiments.

### 2.2. Cell Viability Assay

The rFb were trypsinised and placed in flat-bottomed 96-well plates at an initial density of 5000 cells per well. After 24 h of incubation, the medium was changed to VH298 (purchased from Tocris Bioscience, Bristol, UK; cat. no. 6156)—containing medium at different dosages (0 *μ*M, 10 *μ*M, 30 *μ*M, 100 *μ*M, and 200 *μ*M). rFb were incubated at 37°C for 48 h. The proliferation was determined by the Cell Counting Kit-8 (CCK-8) assay. After incubation, rFb were treated with the CCK-8 solution at a final concentration of 10% for 2 h at 37°C, followed by measurement of absorbance at 450 nm using a microplate reader.

### 2.3. Western Blot

The rFb were trypsinised and placed in 6-well plates. After 24 h of incubation, in some plates, the medium was replaced with different doses of VH298 (0, 10, 30, 100, and 200 *μ*M) or DMOG (500 *μ*M), and the plates were further incubated for 6 h; in other wells, the medium was changed to a medium containing 200 *μ*M VH298, and the plates were incubated for different times (0, 0.5, 2, 6, 24, and 48 h). The cells were lysed in radioimmunoprecipitation assay lysis buffer, and protein concentration was determined using the bicinchoninic acid assay. Protein samples of equal quantity were separated by SDS-PAGE and transferred to the polyvinylidene difluoride membrane on ice. The membrane was subsequently blocked with 5% skimmed milk in TBST buffer (Tris-buffered saline with 0.05% Tween-20) for 1 h at 25°C, followed by incubation with the primary antibodies (HIF-1*α*, CST #3716, 1 : 1000; hydroxy-HIF-1*α*, CST #3434, 1 : 1000; HIF-2*α*, Abcam #Ab20654, 1 : 1000; and *β*-actin, CST #2118, 1 : 2000) overnight at 4°C. After a thorough rinse with TBST, the membrane was hybridised to a horseradish peroxidase-conjugated secondary antibody (anti-rabbit, CST #7074, 1 : 5000) for 1 h at room temperature and then rinsed with TBST again for 30 min. The enhanced chemiluminescence (ECL; Millipore, Billerica, MA) method was employed to visualise the final blots, with *β*-actin as the loading control.

### 2.4. RNA Extraction and Quantitative Real-Time PCR

After treatment of rFb with FM containing different doses of VH298 (0 *μ*M, 10 *μ*M, 30 *μ*M, 100 *μ*M, and 200 *μ*M) for 24 h, total cellular RNA was extracted with the RNA Mini Kit (Invitrogen) and reverse transcribed into cDNA with an M-MLV reverse transcriptase (Invitrogen) according to the manufacturer's instructions. Real-time PCR was performed using the StepOnePlus Real-Time PCR System (Applied Biosystems). The reaction conditions consisted of 10 *μ*l reaction volumes with 1 *μ*l diluted cDNA template, 5 *μ*l SYBR-Green Master Mix (2×), 3.4 *μ*l PCR-grade water, and 0.6 *μ*l of each primer (10 *μ*M). The amplification procedure was carried out as follows: initial denaturation at 95°C for 5 min, followed by 40 cycles of 95°C for 15 s and 60°C for 60 s. The sequences of forward and reverse primers are shown in Table 1. The relative quantification of gene expression was analysed using the values of 2^–ΔΔCT^, normalised against the *β*-actin expression level.

### 2.5. Scratch Test

rFb were collected and seeded in a 6-well plate (10^6^ cells/well). Under each well, marks were drawn for locating the photographed region. When cells grew to confluence, FM was replaced by 1% foetal bovine serum (FBS) containing different doses of VH298 (0 *μ*M, 10 *μ*M, 30 *μ*M, 100 *μ*M, and 200 *μ*M) for 24 h. Using a ruler, a line was marked on each well with the 200 *μ*l pipette tip vertically. To remove cellular debris, the cells were washed thrice with PBS (2 ml). The cells were incubated in FM containing 1% FBS and the same dose of VH298, as mentioned earlier, at 37°C in 5% CO_2_ for 48 h. Photos were taken at 0, 24, and 48 h. Six lines were marked in each group, and two regions on each line were photographed; the scratch area was measured using ImageJ software (Rawak Software Inc., Germany). Cell migration rate (%) = (1 − scratch area/original scratch area) × 100.

### 2.6. hUVEC Tube Formation Assay

Matrigel matrix (BD Biosciences) was used to evaluate capillary-like tube formation in hUVEC in vitro. Briefly, hUVEC were incubated in 6-well plates with ECM containing different doses of VH298 (0 *μ*M, 10 *μ*M, 30 *μ*M, 100 *μ*M, and 200 *μ*M) for 24 h. Matrigel was thawed on ice; 120 *μ*l Matrigel was placed in precooled 48-well plates and incubated at 37°C for 45 min to solidify. hUVEC (5 × 10^4^/well) were resuspended in 250 *μ*l ECM and placed on the Matrigel. After 6 h incubation at 37°C in an atmosphere of 5% CO_2_, tubular structures of hUVEC were observed in the Matrigel under a phase-contrast microscope. The number of meshes, length of total segments, and number of isolated branches were calculated in four randomly chosen fields at 10x magnification per well using ImageJ software (Rawak Software Inc., Germany).

### 2.7. Induction of Hyperglycaemia and the Wound Model

The rat model with hyperglycaemia was induced by intraperitoneal injection of streptozotocin (Sigma-Aldrich) at 65 mg/kg, as described previously [11]; forty-eight male Sprague-Dawley rats were induced accordingly. Fourteen days after streptozotocin injection, blood sample was obtained from tail veins, and blood glucose levels were measured using a glucometer. Rats with blood glucose levels above 30 mmol/l were adopted for further experiments. All mice were anesthetized with pentobarbital, and their backs were shaved and sterilized. A full-thickness excisional wound (Φ8mm) was created on the dorsal back of each rat using a biopsy punch (8 mm, Acuderm Inc., Fort Lauderdale, FL). Twenty-four rats were treated with 30 *μ*M VH298 in PBS (100 microlitres) by local injection, every three days, whereas the others were treated with PBS (100 microlitres) as the control. For avoiding secondary injury, in local injection, needles should pierce into the normal skin contiguous to the wound area and go through subcutaneous tissue to the central point of the wound area. The wounds were photographed immediately and at postoperative days 3, 7, 14, and 21, together with a ruler close to the wound area for comparison.

### 2.8. Histology Staining

At postoperative days 7, 14, and 21, eight rats from each group were sacrificed, and 16 wound tissues were harvested. All tissues were initially fixed in 10% formalin for 48 h and embedded into paraffin. Thin 5 *μ*m sections were cut by a rotary microtome (HM 355S, Thermo Fisher Scientific Inc., Germany) around the centre of every wound tissue. After deparaffinisation, haematoxylin-eosin (HE) staining, Masson trichrome staining, and immunohistochemical staining were performed. The HE-stained sections were used to measure the length of the epithelial tongue, epithelial gap, and granulation tissue area in different groups. The Masson trichrome-stained sections were used to measure collagen accumulation, and quantitative analysis was performed using ImageJ software (Rawak Software Inc., Germany), expressed as the ratio of the area (blue area in granulation tissue/total granulation tissue area).

### 2.9. Immunohistochemical Staining

Immunohistochemical staining was performed using a standard protocol as previously reported [12]. Sections were treated with primary antibodies against rat CD31 (Abcam, 1 : 500, ab119339), HIF-1*α* (CST, 1 : 1000, #3716), hydroxy-HIF-1*α* (CST, 1 : 1000, #3434), and VEGF-A (Servicebio, 1 : 1000, GB11034) overnight at 4°C; a horseradish peroxidase-streptavidin detection system (Dako) was used, followed by counterstaining with haematoxylin. CD31-positive cell clusters were counted as described in the previous study [13]. In brief, 10 regions of interest at the same size (squares about 250 *μ*m on a side) in granulation tissue in each specimen were included for counting CD31-positive cell clusters, and microvessel density was expressed as CD31-positive cell cluster number per square millimeter. The positive-stained area after staining of HIF-1*α*, hydroxy-HIF-1*α*, and VEGF-A in the whole wound area per specimen in three sequential sections (50 *μ*m, 150 *μ*m, and 250 *μ*m) per rat in each group was measured, compared, and expressed as the ratio of the area.

### 2.10. Statistical Analysis

All quantitative data were analysed using GraphPad Prism 6 software for Windows (GraphPad Software). Unpaired *t*-test was used for comparison of mean values with *P* < 0.05, which was considered statistically significant.

## 3. Results

### 3.1. HIF-1*α* and Hydroxy-HIF-1*α* in rFb Accumulated in the Presence of VH298 in a Time- and Dose-Dependent Manner

**Figure 1:**
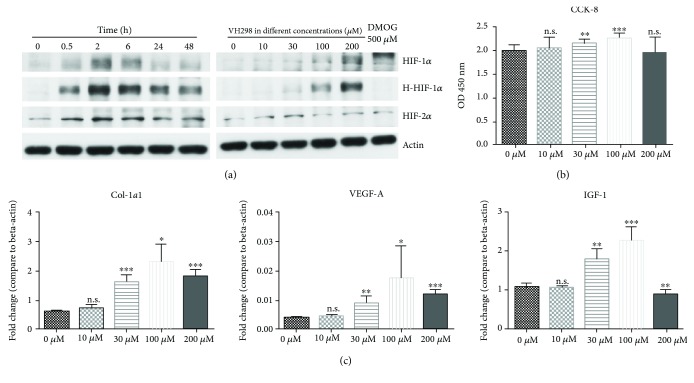
Protein concentration of HIF-1*α*, HIF-2*α*, and hydroxy-HIF-1*α*, cell proliferation, and multiple gene expressions were related to the VH298 dose. (a) After VH298 or DMOG treatment, protein levels in rFb were detected by immunoblots. Protein concentration of HIF-1*α*, HIF-2*α*, and hydroxy-HIF-1*α* in rFb increased gradually along with VH298 concentration, and DMOG only upregulated protein levels of HIF-1*α* and HIF-2*α*, but not hydroxy-HIF-1*α*, after 6 h treatment. 200 *μ*M VH298 increased the HIF-1*α*, HIF-2*α*, and hydroxy-HIF-1*α* protein levels up to 2 h and was followed by a decrease. (b) Cell viability of rFb was evaluated by the CCK-8 assay. VH298 promoted cell proliferation at doses of 30 *μ*M and 100 *μ*M. Graphs represent mean ± SD (VH298-treated vs. control) (*n* = 12). (c) *Col1-α1*, *VEGF-A*, and *IGF-1* gene expressions in rFb were detected by quantitative real-time PCR after treatment with VH298 at different doses, and 30 *μ*M and 100 *μ*M were the most stable doses for upregulation of gene expression. Graphs represent mean ± SD (VH298-treated vs. control) (*n* = 6). ^∗^*P* < 0.05, ^∗∗^*P* < 0.01, and ^∗∗∗^*P* < 0.001; OD: optical density.

Western blot could detect the protein levels of HIF-1*α*, HIF-2*α*, and hydroxy-HIF-1*α*. For 6 h incubation at different doses of VH298, a higher dose of VH298 (up to 200 *μ*M) caused more accumulation of HIF-1*α*, HIF-2*α*, and hydroxy-HIF-1*α* proteins, whereas DMOG (500 *μ*M) caused only HIF-1*α* and HIF-2*α* accumulations. At 200 *μ*M, VH298 caused an increase in the levels of HIF-1*α*, HIF-2*α*, and hydroxy-HIF-1*α* up to 2 h, followed by a decrease (Figure 1(a)).

### 3.2. VH298 Promoted Cell Viability

To investigate the effect of VH298 on cell viability, the CCK-8 assay was performed; results revealed that 30 *μ*M and 100 *μ*M of VH298 promote cell proliferation, while 10 *μ*M and 200 *μ*M of VH298 have no significant effect on cell proliferation (Figure 1(b)).

### 3.3. VH298 Upregulated mRNA of Essential Factors for Wound Healing

Quantitative real-time PCR was performed to evaluate the mRNA level of *Col1-α1*, *VEGF-A*, and *IGF-1*. Compared to the control group (0 *μ*M VH298), 30 *μ*M, 100 *μ*M, and 200 *μ*M VH298 could upregulate mRNA levels of *Col1-α1* and *VEGF-A* while 10 *μ*M VH298 had no significant effect. Relative to the control group, 30 *μ*M and 100 *μ*M upregulated the mRNA level of *IGF-1*, 200 *μ*M downregulated the mRNA level of *IGF-1*, and 10 *μ*M showed no significant effect (Figure 1(c)).

### 3.4. VH298 Promoted rFb Migration

**Figure 2:**
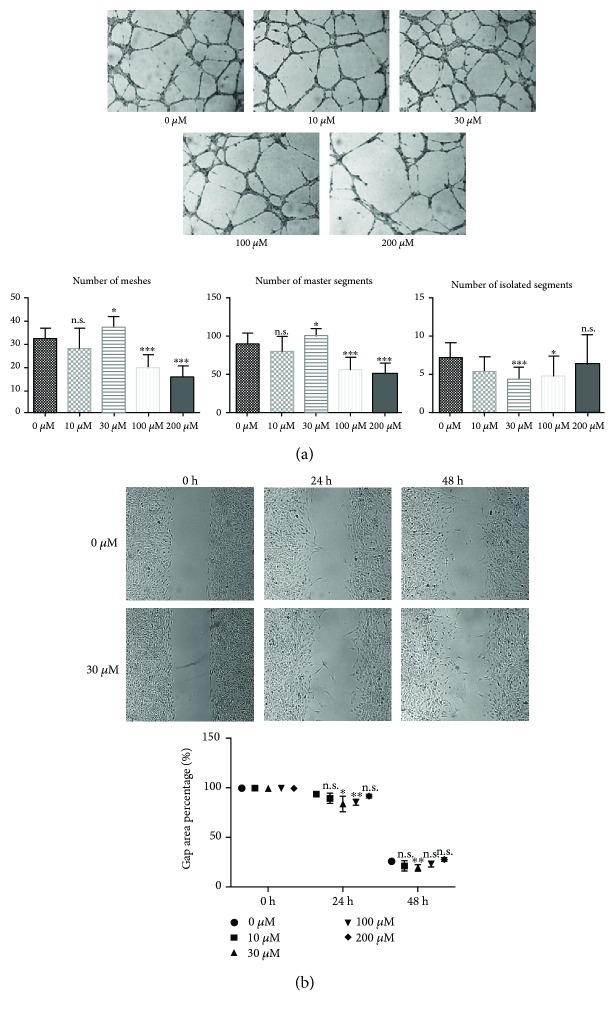
In vitro angiogenesis assay and simulation of wound healing. (a) Angiogenesis measured by the tube formation assay using hUVEC. Tube formation results showed that 30 *μ*M VH298 formed most meshes and master segments and least isolated segments, thereby suggesting that VH298 promotes angiogenesis at low doses but suppresses angiogenesis at high doses, basing on 100 and 200 *μ*M VH298 harming most meshes and master segment formation. Graphs represent mean ± SD (VH298-treated vs. control) (*n* = 6). (b) Scratch test using rFb. 30 *μ*M VH298 significantly accelerated rFb migration after 24 and 48 h observations, and 100 *μ*M VH298 accelerated rFb migration statistically significant only after 24 h observation. Graphs represent mean ± SD (VH298-treated vs. control) (*n* = 6). ^∗^*P* < 0.05, ^∗∗^*P* < 0.01, and ^∗∗∗^*P* < 0.001. n.s.: not significant.

The scratch test was performed to simulate the wound healing process in vitro. The results suggested that VH298 accelerated the migration of rFb. The remaining scratching area was significantly smaller in 30 *μ*M and 100 *μ*M VH298 than in 0 *μ*M after 24 h and smaller in 30 *μ*M VH298 than in 0 *μ*M at 48 h postscratching, whereas the other doses of VH298 have no significant effects on rFb migration compared to 0 *μ*M at the same observation point (Figure 2(b)).

### 3.5. VH298 Resulted in Biphasic Effects on Tubule Formation of hUVEC

We applied the tube formation assay to detect the effect of VH298 on angiogenesis using hUVEC. After preincubation at different doses of VH298 for 24 h, tube formation results showed that 30 *μ*M induced most meshes and master segments and least isolated segments. However, a high dose of VH298 (100 and 200 *μ*M) could severely disturb meshes and master segment formation (Figure 2(a)).

### 3.6. Local Injection of VH298 Accelerated Wound Healing in Rats with DM

**Figure 3:**
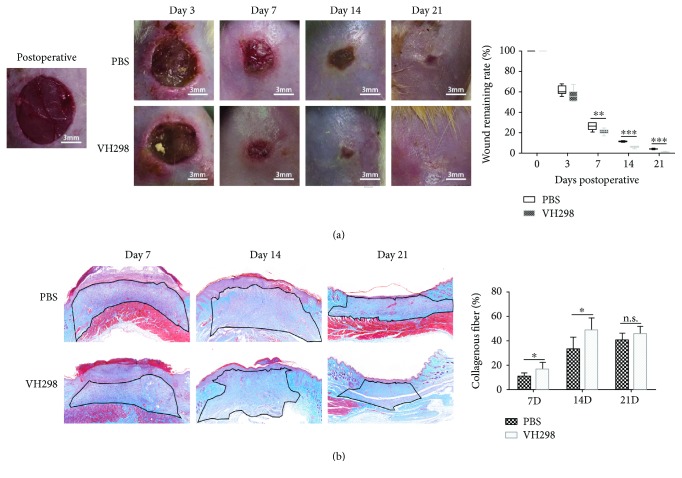
Wound healing rate and collagen accumulation in wound tissue. (a) The remaining wound area was measured after photography. VH298-treated wounds healed much faster compared to PBS-treated wounds. Graphs represent mean ± SD (VH298-treated vs. PBS-treated) (*n* = 8). (b) Masson trichrome staining showed more collagen deposition (blue) in the granulation tissue (black line-circled area), in which quantitative measurement was applied, and the collagen deposition ratio was significantly increased in the VH298-treated group compared to the PBS-treated group at the early and middle stages (7 days and 14 days). Graphs represent mean ± SD (VH298-treated vs. PBS-treated) (*n* = 8). ^∗^*P* < 0.05, ^∗∗^*P* < 0.01, and ^∗∗∗^*P* < 0.001. D: day; n.s.: not significant.

All the photographs of wounds were processed by Photoshop software, and the wound areas were measured and expressed as the remaining wound rate (wound area/initial wound area). VH298-treated wounds healed much faster than the PBS-treated wounds at postoperative days 7, 14, and 21, and no significant effect in the very early stage (3 days) was observed (Figure 3(a)).

### 3.7. Histological Analysis

**Figure 4:**
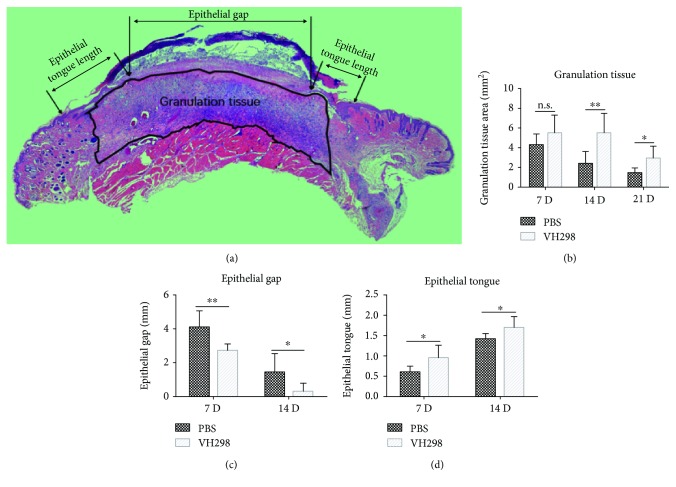
Indicators of wound healing were measured by HE staining. Compared to the PBS group, longer epithelial tongues and shorter epithelial gaps were shown in the VH298 group at days 7 and 14, and larger granulation tissues were seen at days 14 and 21. Graphs represent mean ± SD (VH298-treated vs. PBS-treated) (for granulation tissue and epithelial gap, *n* = 8; for epithelial tongue, *n* = 16). ^∗^*P* < 0.05 and ^∗∗^*P* < 0.01. D: day; n.s.: not significant.

HE staining showed a longer epithelial tongue and thinner epithelial gap in VH298-treated wounds at postoperative days 7 and 14 and a larger granulation tissue area at postoperative days 14 and 21 (Figure 4). Masson trichrome staining suggested more collagen generation in the granulation tissue in VH298-treated wounds at postoperative days 7 and 14 and no significant effect in the late stage (21 days) (Figure 3(b)).

**Figure 5:**
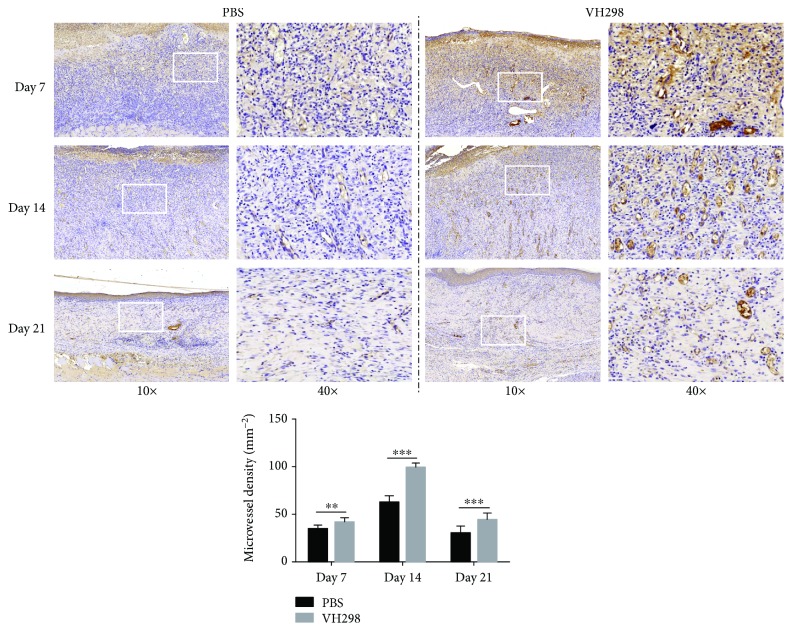
Immunohistochemical staining of CD31 in the wound area. 10x images showed more CD31-positive area (brown) in the VH298-treated wound, and 40x images showed more CD31-positive microvessels (brown) in the VH298-treated wound especially at the middle stage (14 days) of the wound healing process. Quantitative analysis showed more CD31-positive clusters in the VH298-treated group at all three time points. Graphs represent mean ± SD (VH298-treated vs. PBS-treated) (*n* = 8). D: day. ^∗∗^*P* < 0.01 and ^∗∗∗^*P* < 0.001.

**Figure 6:**
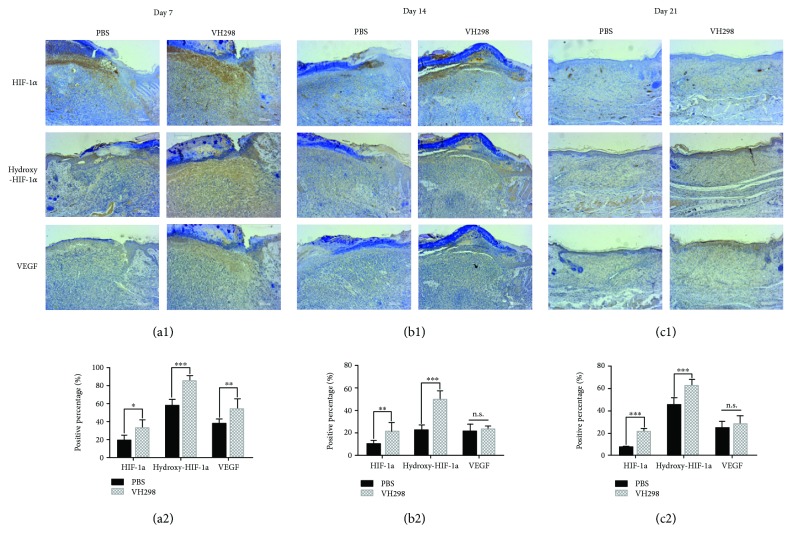
Immunohistochemical analysis reflecting the activation of the HIF signalling pathway following VH298 treatment. (a1–c1) VH298 treatment significantly enlarged HIF-1*α*-, hydroxy-HIF-1*α*-, and VEGF-A-positive areas (brown) compared to PBS treatment. (a2–c2) Values expressed as the positive percentage of each group. Graphs represent mean ± SD (VH298-treated vs. PBS-treated) (*n* = 8). ^∗^*P* < 0.05 and ^∗∗^*P* < 0.01. n.s.: not significant.

Immunohistochemical staining and quantitative analysis showed more CD31-positive cell clusters in the wound healing area in the VH298-treated group than in the PBS-treated group (Figure 5), suggesting better angiogenesis in VH298-treated wounds. Immunohistochemical staining also confirmed more HIF-1*α*-positive, hydroxy-HIF-1*α*-positive, and VEGF-A-positive cells, suggesting the activation of the HIF signal pathway after VH298 treatment (Figures 6(a1)–6(c1) and 6(a2)–6(c2)).

## 4. Discussion

The aim of this study was to investigate the effect of the VHL protein inhibitor VH298 on wound healing, both in vitro and in a rat model with hyperglycaemia. In the present study, we demonstrated that (1) VH298 can activate the HIF-1 signalling pathway by stabilizing both forms of HIF-1*α* both in vivo and in vitro, (2) VH298 promotes rFb variability, migration, and synthesis of collagen and regulatory cell factors, (3) VH298 improves the angiogenesis of hUVEC, and (4) local injection of VH298 accelerates wound healing in a STZ-induced hyperglycaemic rat model. Therefore, we believe that VH298 injection is a potent method to improve wound healing in cases with diabetes.

DM is a complex disorder; wound healing has been found to be much slower in patients with DM than in others, even if diabetes is well under control, probably due to the impaired functions of multiple cells, macrophages, fibroblasts, endothelial cells, keratinocytes, etc. Therefore, enhancing the function of cells involved in wound healing may be considered to accelerate wound healing in DM. Wound healing requires the integration of complex cellular and molecular events in successive phases of inflammation, granulation tissue formation, and reepithelialisation. In particular, dermal fibroblasts play essential roles in the repair of skin wounds through remodelling of the wound bed by the synthesis of new extracellular cell matrix and growth factors and formation of thick actin bundles [14]. Impaired wound healing in patients with diabetes is a well-documented phenomenon, and aberrant fibroblast function contributes to this process [10, 15, 16]. Therefore, improvement of the function of fibroblasts is important in wound healing.

Besides, Catrina et al. first noticed that high glucose/hyperglycaemia impaired HIF-1*α* protection under hypoxia both in vitro and in human diabetic ulcer and pointed out the molecular mechanism connecting hyperglycaemia and hypoxia sensitivity [3]. Mace et al. first noticed that compared to nondiabetic, HIF-1*α* expression was markedly decreased in skin wounds of diabetic mice [4], and the therapeutic potential of HIF-1*α* stabilization for diabetic wound healing has been first shown by Botusan et al. [9]. And Ruthenborg et al. first systematically discussed the potential for the use of PHD inhibitors to treat tissue injuries and wounds [17]. Numerous studies demonstrated that PHD-targeted treatment, such as using DMOG or designed compounds, e.g., FG-4592, had satisfactory outcomes in wound healing via the HIF-1 signalling pathway [8, 18–22]. However, the upregulated HIF-1 via VHL inhibition remained largely unknown. Besides, procollagen maturation is well known to be required in a series of hydroxylation processes relying on lysyl hydroxylases, which also contain Fe(II) in their catalytic centre and also need *α*-ketoglutarate as a cosubstrate [23]. Therefore, PHD inhibitors also affect functions of lysyl hydroxylases and thus disorder the collagen accumulation in the wound area despite the upregulated collagen genes, thereby suggesting that VHL inhibition might be a more consummate way to activate the HIF-1*α* pathway for the treatment of wound in cases with DM.

In the present study, we focused on activating the HIF-1 signal pathway in rFb by VH298. After VH298 treatment with different doses or for different times, HIF-1*α* and hydroxy-HIF-1*α* protein levels in rFb were significantly upregulated in a dose- and time-dependent manner. Immunohistochemical staining results also showed activation of the HIF-1 signalling pathway. Taken together with the well-characterized mode of action of VH298 [7], we suggested that VH298 effectively activates the HIF-1 signalling pathway theoretically by inhibiting hydroxy-HIF-1*α* ubiquitination. mRNA levels of *VEGF-A* and *IGF-1* were also found upregulated in our study. *VEGF-A* and *Col1-α1* are well-known downstream factors of HIF-1 [18–21], whereas *IGF* is regarded as a regulatory factor of HIF-1*α* previously [24]. Even though no previous study has proven IGF-1 as a downstream target of HIF-1, taken together with our findings, we assume a bidirectional regulation of HIF-1*α* and IGF-1 possibly through some unknown mechanisms.

Unlike HIF-1*α* being highly conserved, HIF-2*α*, a HIF-1*α* paralog, has not been found existing in invertebrates [25]. HIF-2*α* was found playing an important role in erythropoiesis, vascularization, and pulmonary development in vertebrates [26] and in regulating erythropoiesis [27]. In the current study, we used VH298 to activate HIF-1 by inhibiting VHL protein, in which HIF-2*α* was also upregulated, which can be proven by the immunoblot results. Even though rare findings proved the function of HIF-2*α* on the wound healing process, we were not supposed to ignore but to beware the potential effects contributing to the outcomes in the current study, for the complicated cellular signalling network.

Despite our data confirming the significantly enhanced functions of rFb and hUVEC by VH298, the microenvironments in vivo are highly dynamic, and the VH298 content likely differs from that in vitro; it will be interesting to examine the properties of VH298 over time or at pre- and postinjection stages. Meanwhile, readers should notice that the animal model we used in the work is an STZ-induced hyperglycaemic model, which is theoretically just a reflection of the acute detrimental effects of hyperglycaemia on wound healing, and it is irresponsible to directly draw parallels with a human situation of years/decades with diabetes. Besides, basing on the biphasic effects of VH298, future research endeavours should emphasise on developing extended-release methods to maintain sustainability of VH298 under a dynamically changing microenvironment, such as in the process of wound healing.

## Figures and Tables

**Table 1:** Sequences of the primers used in quantitative RT-PCR.

Gene	Primer	Sequence
VEGF-A	Forward	5′-CGT CTA CCA GCG CAG CTA TTG-3′
	Reverse	5′-CTC CAG GGC TTC ATC ATT GC-3′

IGF-1	Forward	5′-AAC CTG CAA AAC ATC GGA AC-3′
	Reverse	5′-GCA GCC AAA ATT CAG AGA GG-3′

Col-1*α*1	Forward	5′-TGC TGC CTT TTC TGT TCC TT-3′
	Reverse	5′-AAG GTG CTG GGT AGG GAA GT-3′

*β*-Actin	Forward	5′-TAC CAC TGG CAT TGT GAT GG-3′
	Reverse	5′-AGG GCA ACA TAG CAC AGC TT-3′

## Data Availability

The data used to support the findings of this study are available from the corresponding author upon request.
